# Analytical validation of a homologous recombination deficiency signature (HRDsig) in pan-tumor tissue samples

**DOI:** 10.1371/journal.pone.0336940

**Published:** 2025-11-17

**Authors:** Wenshu Li, Jeffrey A. Leibowitz, Shuoguo Wang, Louisa Walker, Chang Xu, Kuei-Ting Chen, Alexa B. Schrock, Jason Hughes, Nimesh Patel, Julia A. Elvin, Lauren L. Ritterhouse, Ethan Sokol, Garrett Frampton, Lucas Dennis, Bahar Yilmazel, Brennan Decker

**Affiliations:** Foundation Medicine, Inc., Boston, Massachusetts, United States of America; The University of Queensland Faculty of Medicine, AUSTRALIA

## Abstract

Homologous recombination repair (HRR) is a cellular pathway for high-fidelity double strand DNA break repair that uses the sister chromatid as a guide to ensure chromosomal integrity and cell viability. Deficiency in the HRR pathway (HRD) can sensitize tumors to poly (ADP-ribose) polymerase inhibitors (PARPi) and platinum-based chemotherapy, offering an avenue to identify patients who may benefit from targeted therapies. HRD signature (HRDsig) is a pan-solid-tumor biomarker on the FoundationOne®CDx (F1CDx®) assay that employs a DNA scar-based approach to calculate a score based on copy number features (e.g., segment size, oscillation patterns, and breakpoints per chromosome arm) and does not rely on HRR gene alterations, enabling detection of genomic and epigenetic mechanisms of HRD. After finalizing the HRDsig algorithm, analytical validation was conducted in a CAP-accredited, CLIA-certified laboratory on 278 solid tumor and normal tissue specimens. HRDsig results were compared with an independent HRD biomarker, defined by the presence of a reversion mutation restoring HRR gene function. In this evaluation, 100 HRD-positive and 126 HRD-negative samples showed a positive percent agreement of 90.00% and a negative percent agreement of 94.44%. The limit of detection (LoD) was estimated at 23.04% tumor purity, with the limit of blank (LoB) confirmed as zero in 60 normal tissue replicates. Reproducibility testing on 11 positive and 11 negative samples across multiple labs, reagent lots, and sequencers yielded agreement in 99.49% of positive and 99.73% of negative replicates. HRDsig status remained consistent in the presence of interfering substances, demonstrating 100% concordance in spiked samples. These validation results underscore the high analytical concordance, low false-positive rate, and overall robustness of HRDsig for reliable assessment of homologous recombination deficiency.

## Introduction

Homologous recombination repair (HRR) is a cellular pathway for high-fidelity double strand DNA break (DSB) repair that uses the sister chromatid as a guide to ensure chromosomal integrity and cell viability [1]. Homologous recombination deficiency (HRD) is a phenotypic state in which a cell is unable to effectively repair these breaks using the HRR pathway [2]. HRD has been attributed to the presence or occurrence of genetic and/or epigenetic loss-of-function alterations in genes within the HRR pathway.

HRD is associated with cellular sensitivity to poly (adenosine diphosphate [ADP]-ribose) polymerase (PARP) inhibitors and platinum-based chemo therapies through synthetic lethality [3,4]. Some PARP inhibitors (PARPi) trap PARP on DNA at sites of single-strand breaks, preventing the repair of these breaks and generating DSBs that cannot be accurately repaired in cells with HRD. Platinum agents induce covalent DNA cross-linked lesions which trigger the DNA damage response but cannot be efficiently recognized and repaired in cells with HRD. Therefore, tumors characterized by HRD can be selectively targeted by PARP inhibitors therapies and platinum-based chemotherapies [4–16].

However, patient identification for HRD-driven targeted therapy is a persistent challenge. Although clinical biomarkers for HRD can be defined by inactivating genomic alterations in HRR genes (*e.g., BRCA1/2*) that are predicted to result in the HRD phenotype, genomic alterations in HRR genes may not fully or accurately capture the patient population deriving benefit. *BRCA1* promoter hypermethylation is an epigenetic change that silences expression of BRCA1 protein and is reported to drive an HRD phenotype in ovarian and breast cancers [17] but is not captured through DNA mutation calling [17]. Conversely, monoallelic alterations in HRR genes may be insufficient to lead to an HRD phenotype and, more broadly, there is an unclear association of alterations in HRR genes beyond *BRCA1/2* with an HRD phenotype [18,19]. Alternatively, genomic signatures, which can incorporate features beyond just DNA mutations, represent an emerging orthogonal biomarker as they measure the functional outcome of the HRD phenotype, regardless of the underlying mechanism. In ovarian cancer, signatures such as genome-wide loss of heterozygosity (gLOH) and genomic instability score (GIS) have demonstrated clinical utility [7], but their analytical and clinical validity is unclear in other cancer types.

HRD signature (HRDsig) is a pan-solid tumor biomarker on the FoundationOne®CDx (F1CDx ®) assay. HRDsig is a novel genomic signature that does not rely on HRR gene alterations but rather employs a DNA scar-based approach to calculate a score based on copy number features (*e.g.,* segment size, oscillation patterns, and breakpoints per chromosome arm), enabling detection of genomic and epigenetic mechanisms of HRD. Here, we demonstrate the robust, pan-tumor analytical validity of HRDsig for the identification of HRD positive tumors.

## Methods

### F1CDx® assay

**Fig 1:**
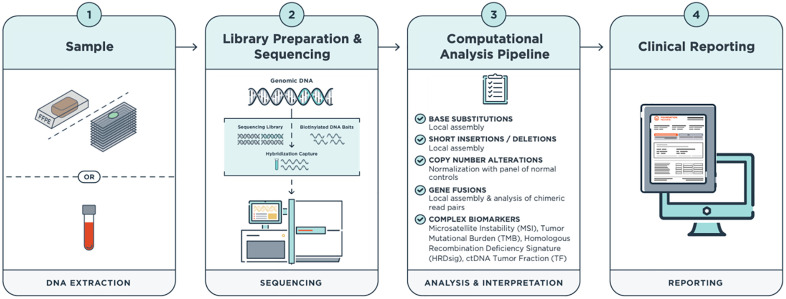
FoundationOne®CDx Test Workflow and Computational Pipeline.

F1CDx® is a Next Generation Sequencing (NGS)-based *in vitro* diagnostic device that targets 324 cancer-related genes. F1CDx® uses hybridization-based capture technology on DNA extracted from FFPE tumor samples for the detection of all major classes of genomic alterations inclusive of substitutions, insertion and deletion alterations, copy-number alterations, and select rearrangements, as well as complex biomarkers, including microsatellite instability, tumor mutational burden. HRDsig is also part of the device, offered as a laboratory professional service, as approved by New York State Department of Health Clinical Laboratory Evaluation Program (CLEP). F1CDx® sequencing and variant calling methods have been described previously [20]. The F1CDx® test workflow and computational pipeline is depicted in Fig 1.

### HRDsig analysis by F1CDx®

HRDsig is a novel genomic signature that employs a DNA scar-based approach to calculate a score based on copy number features, enabling detection of both genomic and epigenetic mechanisms of HRD. The HRDsig algorithm leverages more than 100 copy-number features, including segment size, oscillation patterns, and breakpoints per chromosome arm, with features examined both genome-wide and within the telomeric and centromeric portions of chromosome arms [21,22], which are used as inputs into an extreme gradient boosting (XGB) machine learning model. For training, a set of samples enriched for HRD (cases with biallelic *BRCA1/2* loss-of-function mutations) were labeled as HRD-positive, while a set of samples without mutations in 14 common HRR genes (*BRCA1*, *BRCA2*, *ATM*, *BARD1*, *BRIP1*, *CDK12*, *CHEK1*, *CHEK2*, *FANCL*, *PALB2*, *RAD51B*, *RAD51C*, *RAD51D*, and *RAD54L*) were labeled as HRD-negative. Although the labels are imperfect, these genomic correlates provide sufficient separation between groups to allow XGB modeling to identify HRD-associated scarring patterns. Training and testing were performed on a subset of 282,700 pan-cancer samples profiled with FoundationOne® or F1CDx®. The subset (n = 96,113) includes a variety of cancer types – breast, ovary, pancreas, prostate, and others (the rest of all other cancer types) that can be categorized as HRDsig positive (samples with biallelic *BRCA1/2* alterations) or HRDsig negative and was split 7:3 for training and testing (S1 Table). The XGB model outputs a generally bimodally distributed score between 0 and 1, reflecting the likelihood of a sample being HRD-positive. A cutoff of 0.7 was prespecified for calling a sample HRDsig positive and was established based on 90% sensitivity to detect biallelic BRCA1/2 alterations in cancer types in which HRD has previously been demonstrated to be prevalent (ovary, prostate, pancreas, and breast cancers).

### Samples and materials

Samples used for analytical validation studies consisted of FFPE specimens as well as DNA samples selected from an inventory of residual banked DNA isolated from FFPE tumor specimens. Institutional Review Board (IRB) approval was obtained from the New England IRB prior to use of samples in the described validation studies. HRDsig analytical validation studies were executed between December 23^th^, 2021 and September 19^th^, 2023. The data were accessed on January 6^th^, 2025 for the purpose of evaluation for this manuscript. Authors had no access to information that could identify individual participants during or after data collection.

### Limit of blank analysis

The limit of blank (LoB) describes the highest measurement that is likely to be observed in a blank or negative sample with a predetermined probability. The probability (*i.e.*, the type I error rate or false positive rate) was set at 0.05, according to the industry standard. Five independent HRDsig negative samples were selected: non-tumor DNA derived from normal adjacent tissue from three primary tumor resections (colon adenocarcinoma, skin sarcoma, and uterine endometrial adenocarcinoma) and from two PBMC samples were used to assess the LoB for HRDsig. Each sample was processed across 12 distinct technical replicates for a total of 60 replicates assessed. All 60 replicates passed quality control steps and were considered valid for study analysis; the quality control results for each sample replicates can be found in S1 Data.

The limit of blank for HRDsig positive status calling in blank samples was measured by calculating the false positive rate (FPR). Let $V_{ij}$ be the replicate validity status (1 for valid replicate and 0 for invalid), and $X_{ij}$ denotes the HRDsig positive status (1 for positive and 0 for negative) for $j^{th}$ replicate of sample i, N is the total number of samples tested. The FPR was calculated as:


$$FPR=\;\frac{\sum_{i=\text{1}}^{N}\sum_{j=\text{1}}^{j}X_{ij}{\times V}_{ij}}{\sum_{i=\text{1}}^{N}\sum_{j=\text{1}}^{j}V_{ij}}*\text{1}\text{0}\text{0}\%$$

### Limit of detection analysis

**Fig 2:**
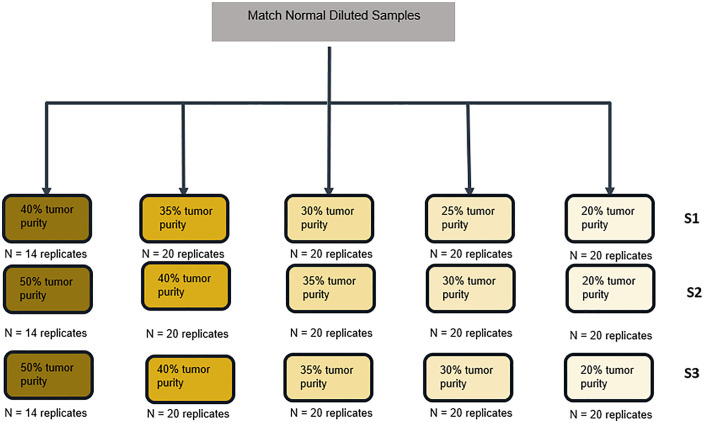
Experimental Design for the LoD Study. Three HRDsig positive breast cancer samples were selected. Sample replicates were titrated to five dilution levels.

The limit of detection (LoD) describes the lowest level at which an analyte (*e.g.*, HRDsig positivity) can be consistently detected [23]. The LoD of the HRDsig biomarker was established using a total of 282 tests across three distinct, HRDsig positive breast cancer specimens, a consensus BRCA- or HRD-associated cancer [22]. The HRDsig algorithm leverages over 100 copy-number features which are produced from F1CDx copy number modeling. Each positive specimen was diluted with matched normal DNA targeting five discrete dilution and tumor purity (TP) levels with multiple replicates per level (Fig 2). Copy number modeling methods use SNP allele frequencies from the sample, so dilution with matched normal is necessary for accurate allele frequency quantification [20]. Replicates that passed quality control steps were considered valid and were included in the analysis. Among the 282 replicates, 15 replicates failed Hybrid Capture quality control and were excluded from analysis (S2 Data).

The LoD for HRDsig positive calls was established as the average adjusted tumor purity determined at the lowest level which achieved at least 95% hit rate in each sample. Empirical hit rate was computed as the number of replicates with positive HRDsig calls divided by the total number of valid replicates at each dilution level. The adjusted tumor purity is a derived value based on the computational tumor purity of an undiluted tumor specimen, adjusted by a dilution factor calculated based on the observed changes in SNP variant allele frequencies (VAFs) in the diluted replicates. LoD was reported as the median adjusted tumor purity across each sample.

### Concordance analysis

There is currently no validated gold standard pan-tumor HRD biomarker that can be used for orthogonal validation in a direct concordance study; as such, inferred truth statuses for both HRD negative and HRD positive states were employed. Although HRD can be caused by mechanisms other than mutations in HRR pathway genes (e.g., epigenetic silencing of *BRCA1* expression [24]), HRD in general, particularly without HRR gene mutations, is rare in pan-cancer samples [18,25]. Therefore, a lack of pathogenic alterations in an extended list of HRR pathway genes (*BRCA1, BRCA2, ATM, BARD1, BRIP1, CDK12, CHEK1, CHEK2, FANCL, PALB2, RAD51B, RAD51C, RAD51D,* and *RAD54L*) was considered as the HRD negative ground truth. Negative truth status was assigned to 130 samples with no detected alterations in any HRR pathway gene among a randomly selected set of samples not used for training or testing of the HRDsig algorithm.

Assignment of HRD positive truth status presented additional challenges in the absence of an acceptable orthogonal assay or clinical outcomes data in this analytical validation study. Loss-of-function reversion mutations (LOF-REV) are alterations that revert a loss-of-function alteration to restore function (*e.g.*, a secondary frameshift mutation restoring the open reading frame of a primary frameshift mutation). LOF-REV in HRR genes are the most common mechanism for resistance to PARPi therapy in HRD tumors due to the strong evolutionary pressure to perform high-fidelity DNA repair in the face of catastrophic DNA damage induced by the synthetic lethality mode of action of these agents [26–28]. LOF-REV are exceedingly rare outside of the context of PARPi therapy and platinum chemotherapy [26,28,29]. This strong enrichment for prior HRD status makes LOF-REV a pragmatic surrogate truth status for HRD status for the purpose of this concordance analysis. Specifically, LOF-REV in *BRCA1, BRCA2, PALB2, BARD1, RAD51D, RAD51C,* and *RAD51B* were considered indicative of prior HRD and were used to assign HRD positive status for this study [30].

101 previously unassessed samples which were not included in the training/testing data of HRDsig algorithm development were identified as containing a LOF-REV alteration in an HRR pathway gene and were used as the ground truth positive sample set. In total, 231 pan-tumor samples were included in the concordance assessment (S3 Data). Two of the 231 samples were excluded from the concordance analysis because they failed quality control metrics during analysis pipeline processing. Among the remaining 229 samples, an additional 3 samples had unknown HRDsig status due to low tumor purity (TP < 10%). These were also excluded from concordance calculations.

Concordance metrics were calculated, as follows.

Let a be the number of samples that are HRDsig positive and have LOF-REV, b be the number of samples that are HRDsig positive and biomarker negative, c be the number of samples that are HRDsig negative and have LOF-REV, d be the number of samples that are HRDsig negative and biomarker negative. The PPA and NPA were defined as:


$$PPA=\frac{a\;}{a+c}\times \text{1}\text{0}\text{0}\%$$

$$\;NPA=\frac{d}{b+d}\times \text{1}\text{0}\text{0}\%$$

The 95% confidence interval was calculated for PPA and NPA using Wilson’s method.

### Precision analysis

The precision analysis evaluated both HRDsig negative and positive reproducibility and repeatability. 22 samples were included, with 11 HRDsig positive and 11 HRDsig negative samples. Pan-tumor representation was targeted, and the analysis included 6 distinct cancer types (ovary, breast, prostate, lung, skin and colon). Intra-run repeatability and reproducibility were assessed across multiple factors including reagent lots, sequencers, laboratory sites, and processing runs, with 2 replicates per sample per run and a total of 792 tests (36 replicates per sample). For a subset of the study samples (N = 10), there were 2 replicates processed per run, using 2 different sequencers using 3 reagent lots across 3 different laboratory sites for a total of 36 replicates per sample; for the remaining study samples, there were 2 replicates processed per run, run in 3 sequencing days across 2 reagent lots across 3 laboratory sites, for a total of 36 replicates per sample (see Table 1). Among the 792 replicates, 4 replicates that failed Library Construction (LC) and 8 replicates that failed HC quality control steps were excluded from analysis. In addition, 25 replicates for sample S12 passed quality control steps but with unknown HRDsig status and were also excluded from analysis. The detailed sample replicate information and quality control metric results and tumor purity are summarized in S4 Data.

**Table 1:** Factorial Design for Samples in Precision Study. Twenty-two samples were processed at 3 different sites, 18 runs, with 2 replicates per run, for a total of 36 replicates per source sample.

Site	Design for sample group1 (N = 12)		Design for sample group2 (N = 10)		Replicates
	Reagent Lot #	Sequencer	Reagent Lot #	Sequencing run #	
1	1	1	1	1	2
1	1	2	1	2	2
1	2	1	1	3	2
1	2	2	2	1	2
1	3	1	2	2	2
1	3	2	2	3	2
2	1	1	1	1	2
2	1	2	1	2	2
2	2	1	1	3	2
2	2	2	2	1	2
2	3	1	2	2	2
2	3	2	2	3	2
3	1	1	1	1	2
3	1	2	1	2	2
3	2	1	1	3	2
3	2	2	2	1	2
3	3	1	2	2	2
3	3	2	2	3	2

The reproducibility and repeatability were calculated as follows:

For sample i, let $\;V_{i}^{p,rep\;k}$ be the replicate validity status, and $X_{i}^{p,rep\;k}$ denotes the HRDsig positive status (1 for positive and 0 for negative) $j^{th}$ replicate of sample i in plate p. $I(X_{i}^{p,rep\;k}=\;X_{i})$ is the indicator function where $I\left(X_{i}^{p,rep\;k}=\;X_{i}\right)=\text{1}$ if $X_{i}^{p,rep\;k}$ is equal to the reference status for sample i ${(X}_{i})$. The reference status for each sample is determined by the majority call rule, i.e., if 50% or above replicates of a sample were HRDsig positive, the reference status for this sample is HRDsig positive. The inter-run precision was quantified by reproducibility and calculated as:


$${Reproducibility}_{i}=\frac{\sum_{p=\text{1}}^{\text{P}}\sum_{k=\text{1}}^{\text{2}}I\left(X_{i}^{p,rep\;k}=\;X\right)\;\times V_{i}^{p,rep\;k}}{\sum_{p=\text{1}}^{\text{P}}\sum_{k=\text{1}}^{\text{2}}\;V_{i}^{p,rep\;k}}$$

### The repeatability was measured as:


$${Repeat}_{i}=\frac{\sum_{i=\text{1}}^{N}\sum_{p=\text{1}}^{P}I\left(X_{i}^{p,rep\;\text{1}}=X_{i}^{p,rep\;\text{2}}\right)\times V_{i}^{p,rep\;\text{1}}{\;\times V}_{i}^{p,rep\;\text{2}}}{\sum_{i=\text{1}}^{N}\sum_{p=\text{1}}^{P}V_{i}^{p,rep\;\text{1}}{\;\times V}_{i}^{p,rep\;\text{2}}}$$

The overall reproducibility and repeatability were calculated by aggregating across all samples.

### Interfering substances analysis

The robustness of HRDsig calling in the presence of potential exogenous and endogenous interfering substances was assessed. DNA samples derived from 11 FFPE source samples (5 HRDsig positive and 6 HRDsig negative) were included. Each sample was processed with 5–12 replicates with a minimum of 1–2 replicates processed as normal and the remainder having a substance spiked in at a pre-specified concentration. Each spike-in substance had a minimum of two replicates processed with the spiked-in substance. In total, 9 interfering substances were assessed (melanin, proteinase K, molecular index barcodes, ethanol, hemoglobin, triglycerides, xylene, conjugated bilirubin, and unconjugated bilirubin). The impact on HRDsig positive calling was assessed for all substances except for conjugated bilirubin. The impact on HRDsig negative calling was assessed for all substances except for xylene. Lastly, six samples with increasing levels of estimated necrosis were used to assess the impact of necrosis with two replicates processed for each sample. As no baseline non-necrotic replicates could be evaluated for these samples, only agreement was calculated. Detailed sample replicate information such as quality control metric results, tumor purity, and spiked-in substances are summarized in S5 Data. Among the 130 total replicates of the 17 samples, 6 replicates failed quality control steps and were excluded from statistical analysis.

The HRDsig status of interest for each sample was determined as the majority status using the replicates tested under control conditions. If the majority HRDsig status could not be determined using control replicates (i.e., only 2 replicates were tested under the testing condition or no control replicates were available to be tested, such as samples with necrosis), the sample was excluded from overall percent agreement analysis but included at the sample level percent agreement analysis.

The percent agreement for HRDsig was assessed for each interferent for each sample. The formula is as follows:


$${PA}_{is}=\;\frac{\sum_{j=\text{1}}^{n_{is}}I(X_{isj}=\;X_{i})\times \;V_{isj}}{\sum_{r=\text{1}}^{n_{is}}V_{isj}}*\text{100}\%$$

Where:

$n_{is}$ is number of replicates of sample i tested for interferent s,

$V_{isj}$ is the validity status of $j^{th}$ replicate sample i tested for interferent s,

$I(X_{isj}=\;X_{i})$ is the indicator function. $I(X_{isj}=\;X_{i})=\text{1}$ if the detected HRDsig in the replicate is equal to the reference HRDsig status; Otherwise, $I(X_{islj}=\;X_{i})=\text{0}$.

The overall percent agreement across all interferent was aggregated across all sample replicates.

## Results

The analytical validation study was performed on 278 unique specimens from a wide range of tumor tissue types. The analytical validity of F1CDx® assay calling HRDsig was demonstrated across multiple analyses reporting LoB, LoD, concordance with surrogate HRD truth biomarkers, precision, and the impact of interfering substances. These findings provide a comprehensive overview of the assay’s accuracy, reproducibility, and robustness under various conditions.

### Assay limit of blank

LoB is defined as the highest measurement result that is likely to be observed for a blank sample with a stated probability – false positive rate or type I error rate. According to the industry standard, false-positive rate <5% was selected. To assess the LoB of HRDsig, 5 samples were examined with 12 replicates for each sample (Table 2). HRDsig positivity was not detected in any of the 60 sample replicates processed and the overall FPR was 0.00% (S1 Data).

**Table 2:** LoB of FoundationOne®CDx Detection HRDsig Positivity in Normal (non-cancer) Tissue Samples.

Analysis	SOURCE ID	False Positive Rate (%)
Overall	Overall	0.00(0/60)
Sample Level	S4	0.00 (0/12)
Sample Level	S5	0.00 (0/12)
Sample Level	S6	0.00 (0/12)
Sample Level	S7	0.00 (0/12)
Sample Level	S8	0.00 (0/12)

All the sample replicates passed quality control metrics and were included in the statistical analysis. No HRDsig positivity was detected in any replicate. As expected for blank (normal) samples with no detectable aneuploidy or HRDsig signal, the pipeline did not output HRDsig scores.

### Limit of detection

**Fig 3:**
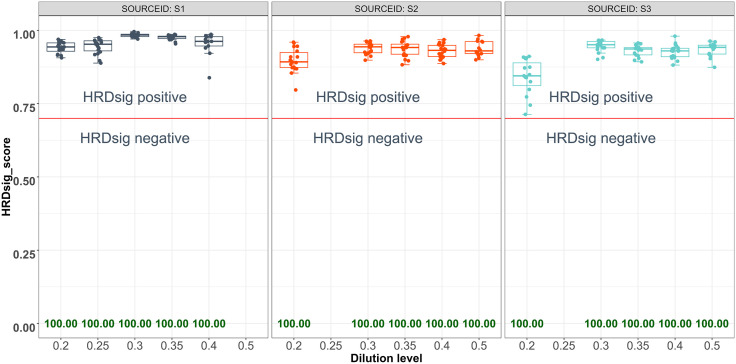
Distribution of HRDsig Score in LoD Study Samples. The hit rate for each dilution level can be found annotated along the x-axis.

To assess LoD, hit rates at each diluent level were calculated for HRDsig positivity and samples were examined with 5 dilution levels of decreasing sample tumor purity (Table 3 and S2 Data). The hit rate for HRDsig positivity was observed as 100% across all five dilution levels for each of the three study samples (Fig 3). The target of 95% hit rate was achieved for each of the three study samples at the lowest dilution level of 20%. Dilutions below 20% were not systematically conducted. The corresponding mean adjusted TP at the targeted 20% dilution level for the three breast cancer samples were 23.04%, 24.51%, and 12.21%. The LoD for HRDsig positive detection by F1CDx® was determined to be the median adjusted TP across the three study samples, 23.04%. The magnitude of the HRDsig scores were plotted in Fig 3, with a larger drop and greater variation in HRDsig score observed when TP was below the study-determined LoD level (23.04%). However, even though scores were lower and had more variability, all replicates were above the 0.7 threshold for positivity.

**Table 3:** LoD of FoundationOne®CDx for Detection HRDsig Positivity.

Sample	Dilution Level	Hit Rate (%) 95% CI [%]	Avg Adjusted %TP
**S1**	**0.2**	**100.00 (20/20) [83.89, 100.00]**	**23.04%**
S1	0.25	100.00 (19/19) [83.18, 100.00]	27.41%
S1	0.3	100.00 (20/20) [83.89, 100.00]	33.05%
S1	0.35	100.00 (20/20) [83.89, 100.00]	39.32%
S1	0.4	100.00 (13/13) [77.19, 100.00]	43.09%
**S2**	**0.2**	**100.00 (18/18) [82.41, 100.00]**	**24.51%**
S2	0.3	100.00 (19/19) [83.18, 100.00]	34.67%
S2	0.35	100.00 (20/20) [83.89, 100.00]	40.14%
S2	0.4	100.00 (20/20) [83.89, 100.00]	44.50%
S2	0.5	100.00 (14/14) [78.47, 100.00]	53.13%
**S3**	**0.2**	**100.00 (15/15) [79.61, 100.00]**	**12.21%**
S3	0.3	100.00 (18/18) [82.41, 100.00]	29.60%
S3	0.35	100.00 (17/17) [81.57, 100.00]	35.52%
S3	0.4	100.00 (20/20) [83.89, 100.00]	40.61%
S3	0.5	100.00 (14/14) [78.47, 100.00]	49.28%

Out of the total 282 testing sample replicates, 15 replicates failed Hybrid Capture quality control and were excluded from analysis. LoD for each source sample was established at the lowest dilution level. The LoD of HRDsig was determined as the median LoD (23.04%) across all three study samples.

### Concordance

There is no established gold standard test for a pan-tumor HRD biomarker and, as a result, genomic alterations well established as associated with the HRD phenotype were leveraged as a surrogate truth for the concordance analysis. HRDsig calling agreement was evaluated in 231 samples against pre-determined surrogate truth statuses for HRD, either LOF-REV for HRD positive status or a lack of any alteration in an HRR pathway gene for HRD negative status (S3 Data).

Detected LOF-REV alterations in HRR genes (reversions in *BRCA1, BRCA2, PALB2, BARD1, RAD51D, RAD51C,* and *RAD51B*) were considered as HRD positive. PPA was calculated across all LOF-REV positive samples with a valid HRDsig status. As shown in Table 4, among the 100 LOF-REV-positive samples with valid HRDsig status, 90 samples were HRDsig positive, hence the PPA was 90.00% (90/100). There were 10 PPA discordant samples in which the sample’s F1CDx® results identified a putative LOF-REV alteration but the HRDsig biomarker was negative. Among these 10 samples, 4 samples had a computational tumor purity (range: 10.08% – 17.37%) below the HRDsig LoD (23.04%). An additional two discordant samples had HRDsig scores just below the positivity threshold of 0.7 (S6 Data). One additional discordant sample was labeled as LOF-REV positive based upon the initial screening criteria but was LOF-REV negative upon re-analysis (a pathogenic frameshift variant originally detected was correctly filtered as an artifact), raising the possibility that the HRDsig positive truth status was incorrectly assigned. S6 Data summarizes detailed data for all discordances.

**Table 4:** Concordance of HRDsig Detection with Established HRD Ground Truth.

	LOF-REV*	Biomarker Negative*	Total
**HRDsig Positive**	90	7	97
**HRDsig Negative**	10	119	129
**Unknown HRDsig status**	1	2	3
**Total**	101	128^#^	229^#^
	PPA = 90.00% (90/100) 95% CI: 82.56%− 94.48%	NPA = 94.44% (119/126) 95% CI: 88.98%− 97.28%	

*Reversion Mutations of biallelic loss of function (LOF-REV) in HRR genes were used to define positive truth status. Negative status was defined as the lack of detection of any alteration in any HRR pathway gene.

# Two biomarker negative samples failed pipeline QC and excluded from analysis. HRDsig status unknown samples were excluded from concordance analysis.

Due to the lack of a gold-standard for defining HRD negative status of a given sample, HRR wild type status was utilized to select a sample population expected to be enriched for HRD negative status. Among the 128 HRD biomarker negative samples, 119 samples were HRDsig negative, 7 samples were HRDsig positive, and the remaining 2 samples had an unknown HRDsig status, resulting in an NPA of 94.44% (119/126). The NPA would be 92.97% (119/128) by including the 2 biomarker negative samples with unknown HRDsig status. There were 7 discordant samples in which the F1CDx® results did not identify the presence of any alteration within genes in the HRR pathway. It is important to note that non-genomic mechanisms may lead to an HRD phenotype, and this population would be expected to present as mutation-negative /HRD-positive and be erroneously assigned to the HRD negative group. Indeed, the majority (4/7) of the discordant samples were from disease ontologies (breast and ovarian cancers) in which epigenetic mechanisms of HRD have been reported to be common. In addition, gLOH findings across all 7 samples demonstrated an elevated gLOH score (≥16%), providing additional support for the true HRD status of the samples. NPA discordances were summarized in S6 Data.

Overall, the results suggest strong positive and negative agreement for HRDsig status calling between our HRDsig and the designated surrogate truth markers.

### Precision

**Fig 4:**
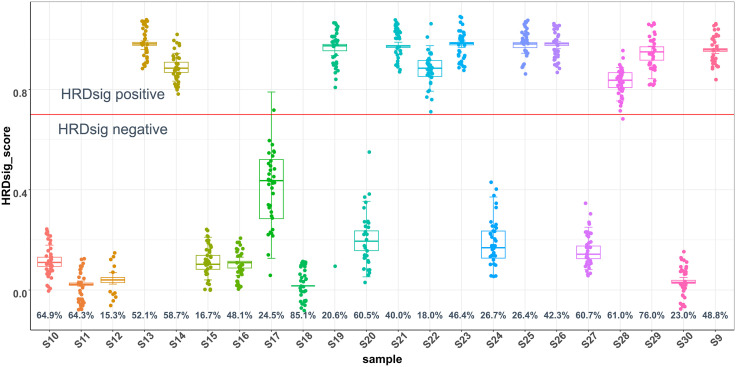
Distribution of HRDsig Score of Precision Study Samples: HRDsig Scores for All Replicates with Valid HRDsig Scores. The average percent tumor purity of each sample was annotated along x-axis.

To assess the inter-run and intra-run precision (reproducibility and repeatability) of the F1CDx® assay for calling HRDsig, 22 samples from 6 different cancer types were selected and processed (S4 Data). The reference status for each sample was established based on the majority call across all replicates. 11 samples had a reference status of HRDsig positive and positive reproducibility was measured in these samples. Negative reproducibility was assessed in the remaining HRDsig negative samples. Nine out of the 11 HRDsig positive samples had a positive reproducibility of 100.00% and only two discordances were found in the remaining 2 samples (one per sample). High negative reproducibility was also observed in the HRDsig negative samples: 10 out of 11 samples had 100.00% negative reproducibility and only 1 discordance was observed in a single sample (Table 5). The assay also demonstrated high repeatability for HRDsig status calling – the overall repeatability was 99.19% (368/371) (Fig 4). HRDsig scores were largely consistent for each sample although some variations were observed, primarily for samples with a median HRDsig score in the intermediate range. The HRDsig algorithm was designed to produce a dichotomized distribution of near binary results (near 0 or near 1), and as a result samples with an intermediate phenotype tend to vary in score more widely. Overall, the results suggest high inter- and intra- run precision of the F1CDx® assay for calling HRDsig.

**Table 5:** Reproducibility and Repeatability of HRDsig Calling.

SOURCEID	DO	Mean Tumor Purity	HRDsig Status*	LoD Category^#^	Reproducibility (%) [95% CI (%)]	Repeatability (%) [95% CI (%)]
S12	Breast	15.35%	Negative		100.00(11/11) [74.12, 100.00]	100.00(2/2)
S11	Breast	64.34%	Negative		100.00(36/36) [90.36, 100.00]	100.00(18/18) [82.41, 100.00]
S10	Breast	64.95%	Negative		100.00(36/36) [90.36, 100.00]	100.00(18/18) [82.41, 100.00]
S15	CRC	16.75%	Negative		100.00(36/36) [90.36, 100.00]	100.00(18/18) [82.41, 100.00]
S16	Lung	48.14%	Negative		100.00(36/36) [90.36, 100.00]	100.00(18/18) [82.41, 100.00]
S17	NSCLC	24.52%	Negative		97.22(35/36) [85.83, 99.51]	94.44(17/18) [74.24, 99.01]
S18	Ovary	85.10%	Negative		100.00(36/36) [90.36, 100.00]	100.00(18/18) [82.41, 100.00]
S20	Ovary	60.54%	Negative		100.00(32/32) [89.28, 100.00]	100.00(16/16) [80.64, 100.00]
S24	Prostate	26.67%	Negative		100.00(35/35) [90.11, 100.00]	100.00(17/17) [81.57, 100.00]
S27	Prostate	60.67%	Negative		100.00(36/36) [90.36, 100.00]	100.00(18/18) [82.41, 100.00]
S30	Skin melanoma	23.05%	Negative		100.00(34/34) [89.85, 100.00]	100.00(16/16) [80.64, 100.00]
**Overall Negative Reproducibility**					**99.73(363/364)** **[98.46, 99.95]**	
S13	Breast	52.06%	Positive	>1.5x	100.00(33/33) [89.57, 100.00]	100.00(16/16) [80.64, 100.00]
S9	Breast	48.76%	Positive	>1.5x	100.00(35/35) [90.11, 100.00]	100.00(17/17) [81.57, 100.00]
S14	Breast	58.74%	Positive	>1.5x	100.00(36/36) [90.36, 100.00]	100.00(18/18) [82.41, 100.00]
S21	Ovary	40.03%	Positive	>1.5x	100.00(36/36) [90.36, 100.00]	100.00(18/18) [82.41, 100.00]
S23	Ovary	46.36%	Positive	>1.5x	100.00(36/36) [90.36, 100.00]	100.00(18/18) [82.41, 100.00]
S29	Prostate	76.00%	Positive	>1.5x	100.00(36/36) [90.36, 100.00]	100.00(18/18) [82.41, 100.00]
S28	Prostate	61.01%	Positive	>1.5x	97.22(35/36) [85.83, 99.51]	94.44(17/18) [74.24, 99.01]
S26	Prostate	42.32%	Positive	>1.5x	100.00(36/36) [90.36, 100.00]	100.00(18/18) [82.41, 100.00]
S25	Prostate	26.36%	Positive	1−1.5x	100.00(36/36) [90.36, 100.00]	100.00(18/18) [82.41, 100.00]
S19	Ovary	20.58%	Positive	<LoD	97.14(34/35) [85.47, 99.49]	94.12(16/17) [73.02, 98.95]
S22	Ovary	18.03%	Positive	<LoD	100.00(36/36) [90.36, 100.00]	100.00(18/18) [82.41, 100.00]
**Overall Positive Reproducibility**					**99.49(389/391) [98.15, 99.86]**	
**Overall Repeatability**						**99.19(368/371) [97.65, 99.72]**

Inter-run reproducibility and intra-run repeatability were evaluated for each sample and all samples combined. The 95% confidence intervals were calculated for reproducibility and repeatability with more than 10 sample replicates using Wilson’s method.

### Interfering substances

The impact of potentially interfering substances on HRDsig status calling in the F1CDx® assay was evaluated in 17 samples (S5 Data). For each sample, HRDsig status was determined based on the majority call across replicates tested under control conditions. Necrosis-interfering samples were excluded from the primary analysis results due to the lack of control or baseline replicates, resulting in an undeterminable ground truth for these samples.

The overall percent agreement of HRDsig status across all 112 valid replicates was 100% (95% CI: [96.88%, 100%]). Sample-level percent agreement results for HRDsig status are summarized in Table 6. In the additional exploratory investigation of the impact of necrosis, only one discordant result was observed at the highest necrotic level (50%). It is possible that the extent of necrosis was the driving factor in this discrepancy. However, it is important to note that one of the two replicates had an HRDsig score very close to the positivity threshold of 0.7 (0.6774), while the other replicate was HRDsig positive (above the 0.7 cutoff).

**Table 6:** Interfering Substances Analysis. Sample-level Percent Agreement of HRDsig Status for Each Substance.

Source Sample ID	Interfering Substance	Concentration	% Agreement
S262	Conjugated Bilirubin	0.8g/L	100.00(2/2)
S262	DMSO Control	NA	100.00(2/2)
S262	Hemoglobin	0.8g/L	100.00(2/2)
S262	Normal Control	NA	100.00(4/4)
S262	Triglycerides	148mmol/L	100.00(2/2)
S263	Conjugated Bilirubin	0.8g/L	100.00(2/2)
S263	Dimethyl sulfoxide Control	NA	100.00(2/2)
S263	Hemoglobin	0.8g/L	100.00(2/2)
S263	Normal Control	NA	100.00(4/4)
S263	Triglycerides	148mmol/L	100.00(2/2)
S264	Conjugated Bilirubin	0.8g/L	100.00(2/2)
S264	Dimethyl sulfoxide Control	NA	100.00(2/2)
S264	Hemoglobin	0.8g/L	100.00(2/2)
S264	Normal Control	NA	100.00(4/4)
S264	Triglycerides	148mmol/L	100.00(2/2)
S265	Ethanol	5% of elution buffer volume	100.00(2/2)
S265	Molecular Index Barcodes	30% of MIB index volume	100.00(2/2)
S265	Normal Control	NA	100.00(4/4)
S265	Proteinase K	0.08mg/ml	100.00(2/2)
S266	Ethanol	5% of elution buffer volume	100.00(2/2)
S266	Melanin	0.2μg/ml	100.00(2/2)
S266	Molecular Index Barcodes	30% of MIB index volume	100.00(4/4)
S266	Normal Control	NA	100.00(2/2)
S266	Proteinase K	0.08mg/ml	100.00(2/2)
S267	Dimethyl sulfoxide Control	NA	100.00(2/2)
S267	Normal Control	NA	100.00(1/1)
S267	Unconjugated Bilirubin	0.2g/L	100.00(2/2)
S268	Ethanol	5% of elution buffer volume	100.00(2/2)
S268	Molecular Index Barcodes	30% of MIB index volume	100.00(2/2)
S268	Normal Control	NA	100.00(4/4)
S268	Proteinase K	0.08mg/ml	100.00(2/2)
S269	Hemoglobin	2mg/ml	100.00(2/2)
S269	Normal Control	NA	100.00(1/1)
S269	Triglycerides	37 mmol/L	100.00(2/2)
S269	Xylene	0.0001%	100.00(2/2)
S270	Necrotic	5%	100.00(2/2)
S271	Necrotic	10%	100.00(2/2)
S272	Necrotic	15%	100.00(2/2)
S273	Necrotic	25%	100.00(2/2)
S274	Necrotic	40%	100.00(2/2)
S275	Necrotic	50%	50.00(1/2)
S276	Ethanol	5% of elution buffer volume	100.00(2/2)
S276	Melanin	0.2μg/ml	100.00(2/2)
S276	Normal Control	NA	100.00(2/2)
S276	Proteinase K	0.08mg/ml	100.00(2/2)
S277	Ethanol	5% of elution buffer volume	100.00(2/2)
S277	Melanin	0.2μg/ml	100.00(2/2)
S277	Molecular Index Barcodes	30% of MIB index volume	100.00(4/4)
S277	Normal Control	NA	100.00(2/2)
S277	Proteinase K	0.08mg/ml	100.00(2/2)
S278	Ethanol	5% of elution buffer volume	100.00(2/2)
S278	Melanin	0.2μg/ml	100.00(2/2)
S278	Molecular Index Barcodes	30% of MIB index volume	100.00(4/4)
S278	Normal Control	NA	100.00(2/2)
S278	Proteinase K	0.08mg/ml	100.00(2/2)

## Discussion

The HRD phenotype is driven by inactivation of one or more HRR genes. However, defining a comprehensive HRD biomarker presents numerous technical, biological, and clinical challenges. These include disagreement in the literature regarding which genes cause HRD when mutated, the fact that both genetic and epigenetic changes can result in HRD, and that monoallelic alterations are not sufficient to lead to an HRD phenotype. Although the F1CDx® HRDsig assay was primarily trained using biallelic LOF alterations in *BRCA1* and *BRCA2* as the ground truth for identifying HRD-positive status [22], it also demonstrates the capability to detect other important mechanisms of HRD. Specifically, HRDsig can identify *BRCA1* promoter hypermethylation in ovarian cancer, a key epigenetic alteration that leads to silencing of the *BRCA1* gene and contributes to HRD [22,31]. This ability to capture non-genomic mechanisms of HRD broadens the scope of the assay beyond just genetic mutations, offering a more comprehensive approach to HRD detection. Additionally, HRDsig strongly enriches for biallelic alterations in *PALB2, BARD1, BRIP1, RAD51C, and RAD51D* [32], which are critical genes in the HRR pathway. These genes are involved in maintaining genomic stability by facilitating DNA double-strand break repair, and their inactivation through biallelic alterations can mimic the HRD phenotype seen in BRCA1/2-deficient tumors. This expanded sensitivity of HRDsig to a range of alterations beyond BRCA1/2 enhances its ability to identify HRD-positive tumors, thus increasing its utility in identifying candidates for HRD-targeted therapies.

The analytical validation of HRDsig consisted of a series of non-clinical studies evaluating the impact of technical variation on the accuracy, sensitivity, specificity, reliability, and robustness of the assay. The study designs for the five analytical studies (LoB, LoD, Precision, Concordance, and Interfering Substances) followed guidelines and perspectives from the United States Food & Drug Administration (FDA) and the Clinical Laboratory Standards Institute (CLSI) [23,33,34].

The LoB study confirmed a low false positive rate for HRDsig calls in normal tissues, with a 0.00% false positive rate (0/60). This demonstrates high analytical specificity, which is essential for achieving clinical specificity. The LoD study established a low minimum tumor purity requirement for HRDsig calling with the F1CDx® assay. The LoD was determined to be 23.04% tumor purity, which represents the median of three study samples (12.21%, 23.04%, and 24.51%). Notably, the assay showed a 100% hit rate at a tumor purity level of 12.21%, indicating the high sensitivity of the F1CDx® assay in detecting HRDsig positivity even below the established LoD. Analysis of real-world HRDsig results by computationally-derived tumor purity (S1 Fig) supported the demonstrated LoD of 23.04%. Significant numbers of HRDsig-positive samples were still detected below this level, indicating the assay still retains sensitivity at lower tumor purities.

Identifying a pan-cancer, validated reference assay for HRDsig orthogonal concordance proved challenging. Currently available HRD assays typically focus on inactivating genomic alterations in HRR genes (*e.g.*, BRCA1/2) or measure scar-based mutational signatures resulting from HRD only in limited cancer indications. The definition of HRD and measurement methods across assays vary significantly [35]. Additionally, most existing HRD assays are designed for specific cancer types (*e.g.*, ovarian cancer), making it difficult to identify an external assay to serve as the reference assay in the concordance study. Genomic signatures represent an important readout for identifying HRD as they represent an outlet for measuring the functional, genomic outcome of the HRD phenotype, regardless of the cause. As a result, we used LOF-REV alterations as a functional readout to confirm prior HRD status in the concordance study. The high concordance rates (PPA = 90.0%, 95% CI: [82.56%, 94.48%]; NPA = 94.44%, 95% CI: [88.98%, 97.28%]) between HRDsig status measured by the F1CDx® assay and the functional readout confirmed the accuracy of HRDsig testing.

To assess the reliability of HRDsig testing on the F1CDx® assay, 22 samples (36 replicates per sample) across multiple tumor types and tumor purity levels were tested in the precision study. Nearly 100% reproducibility and repeatability confirmed both inter-run and intra-run precision. The reproducibility results of 97.14% (34/35) and 100% (35/35) for two samples below the LoD further emphasized the assay’s ability to reliably detect HRDsig, even in low tumor purity samples. Lastly, the 100% concordance in the interfering substance study demonstrated the assay’s robustness, showing minimal impact from interfering substances.

## Limitations

While the results of analytical validation studies herein support the sensitivity, specificity, accuracy, reliability, and robustness of HRDsig, there are a few limitations. First, although HRDsig was developed as a pan-tumor biomarker, the study samples were not representative of all tumor types. Most samples were from tumor types with a higher prevalence of HRD (*e.g.*, ovarian and breast cancer). The HRDsig score distributions from the concordance study cohort (S2 Fig) demonstrated a bimodal distribution and consistently high HRDsig scores among HRDsig-positive samples across the broad range of tumor types included in that analysis. This trend is similar to real world results in a larger cohort [32] where HRDsig scores were bimodally distributed in the range of 0.0–0.3 and 0.8–1.0, and the pre-specified 0.7 cut-off showed broad applicability across different tumor types. Second, the LoD was established based on three breast cancer samples, and it is possible that the LoD for HRDsig may vary across different tumor types. Sample availability for LoD was challenging due to the requirement of using matched normal DNA as diluent. Sourcing both tumor-derived DNA and matched normal DNA, with the additional criterion of HRDsig positivity, limited availability to the three breast cancer samples presented in this work. Furthermore, the LoD study had a 100% hit rate even at the lowest dilution level, raising the likelihood that the actual LoD could be lower than the established 23.04% tumor purity, though reporting the relatively conservative LoD value of 23.04% tumor purity helps reduce the risk of overinterpretation of false negative results. Further exploration of the HRDsig score distribution in the LoD and precision studies supports the supposition that the established value represents a conservative estimate. Lastly, these analytical validation studies were not designed to assess clinical performance, and emerging clinical evidence supporting HRDsig has been investigated in other studies [22,31,32,36]. Additional clinical validation studies across tumor types and in *BRCA* and HRR wildtype populations are needed to further assess the clinical validity of the F1CDx® assay for HRDsig.

## Conclusion

This study presents the analytical validation data for F1CDx® calling HRDsig. The results of this study highlight the accuracy, sensitivity, specificity, reliability, and robustness of the F1CDx® assay in detecting HRDsig status, with strong agreement observed when compared to established ground truth for HRDsig. Another notable finding from this study is the ability of the F1CDx® assay to maintain very high reproducibility for detecting HRDsig status, even in cases with interfering substances or low tumor purity. The findings of this study support the use of the F1CDx® assay to detect HRDsig as a valuable and accurate tool in precision oncology.

## Supporting information

S1 Data One variant per line data for LoB study. (XLSX)

S2 Data One variant per line data for LoD study. (XLSX)

S3 Data One variant per line data for concordance study. (XLSX)

S4 Data One variant per line data for precision study. (XLSX)

S5 Data One variant per line data for interfering substance study. (XLSX)

S6 Data Discordant Samples in Concordance Study. (XLSX)

S1 Table Training and Testing Set for HRDsig Algorithm Development. (XLSX)

S1 Fig Prevalence of HRDsig positive findings across 580,546 tumor samples. (TIF)

S2 Fig HRDsig Score Distribution by Tumor Type for the Concordance Study. (TIF)
