# Supplementary figures and images for: Analytical validation of a homologous recombination deficiency signature (HRDsig) in pan-tumor tissue samples
Source: PLoS One. 2025 Nov 17;20(11):e0336940. doi: 10.1371/journal.pone.0336940 (PMC12622852; doi:10.1371/journal.pone.0336940)

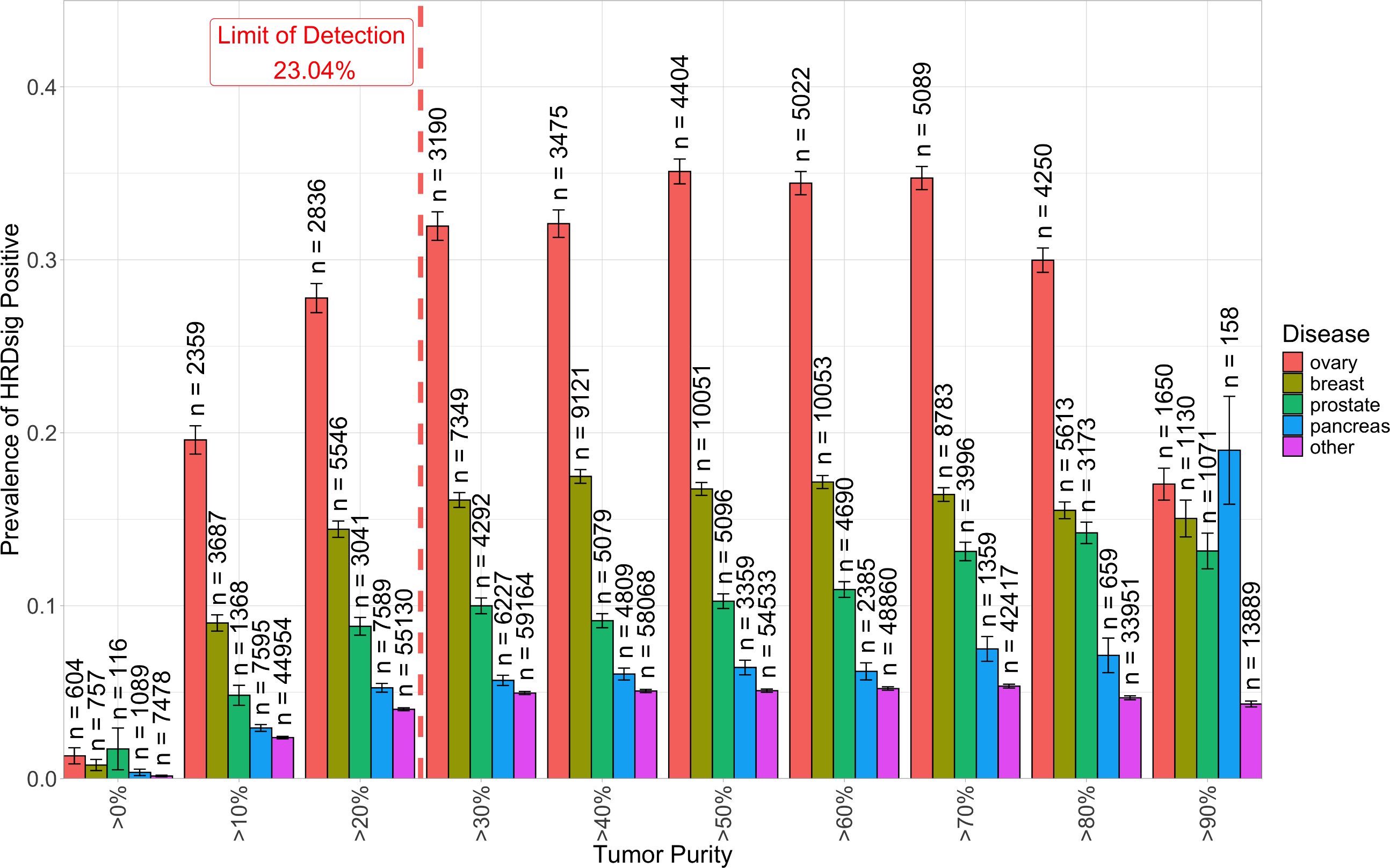

Supplement: S1 Fig — (TIF) [file pone.0336940.s008.tif]

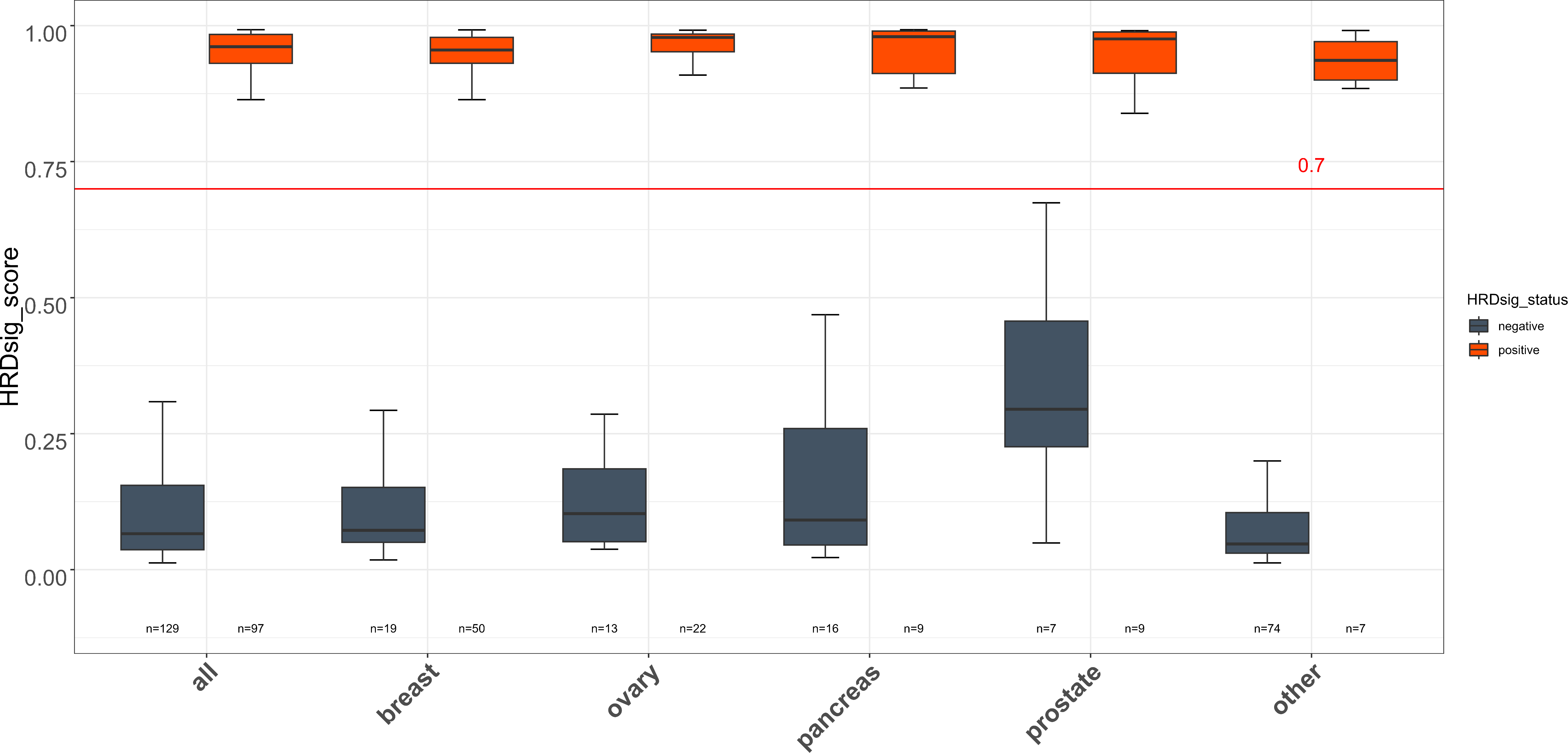

Supplement: S2 Fig — (TIF) [file pone.0336940.s009.tif]
